# Supplementary material for: Natural history of retinitis pigmentosa based on genotype, vitamin A/E supplementation, and an electroretinogram biomarker
Source: JCI Insight. 2023 Aug 8;8(15):e167546. doi: 10.1172/jci.insight.167546 (PMC10445682; doi:10.1172/jci.insight.167546)
Supplement: Supplemental data [file jciinsight-8-167546-s046.pdf]

Supplemental Methods

<u>ERG data processing</u>

Data quality flags produced at the time of data acquisition were used to censor data marked as unreliable. A natural log transformation was applied to normalize the distribution of 30 Hz cone flicker ERG amplitudes. Remaining reliable data from right and left eyes were averaged. The amplitudes were intentionally not adjusted for refractive error, to avoid systemically adjusting the averages of certain genotypes which typically have larger refractive errors. Each trial had two baseline visits for each subject, whose values were averaged into a single visit to produce a dataset with only one set of values per year for each subject. Follow-up visit times since the merged baseline visit were rounded to the nearest year. The rate of decline for subjects with very small baseline ERG amplitudes cannot be accurately determined due to higher test-retest variability (“floor effect"). Therefore for the subset of regression analyses that modeled ERG decline over time, a "higher amplitude cohort" was used with baseline amplitudes of at least 0.68 microvolts. For cross-sectional studies, data points from all subjects were used without filtering for a higher starting amplitude. The baseline cone flicker implicit time (alone, and crossed with time) were rescaled to a mean of zero and used as a predictive factor as described. Implicit times from non-baseline visits were not used in the analysis.

<u>Longitudinal analyses in SAS</u>

A mixed model using SAS version 3.6 was initially used for regression modeling of longitudinal outcomes, to most closely approximate the analysis in the original paper (1). (However, the software used in the SAS statistical analysis package has been upgraded significantly since the early 1990s, and the original mainframe version is no longer available.) A mixed model was used with subject as random variable, and the rounded year as a class variable, "time". The 2x2 factorial treatment design requires additional coding compared to designs with independent treatment arms; dummy variables for

treatment group and treatment group * time were constructed. Treatment groups were: group 1 as A+/E-, group 2 A-/E- (reference), group 3 A+/E+, group 4 A-/E+ , where "+" = "high" and "-" = "trace". (The reference group, which would be the first or last group by convention, was chosen as “group 2” for historical reasons only.) These dummy variables allowed for the use of linear contrasts to model effects of vitamin A and E, as was done for the original study, and as is recommended for 2x2 factorial designs (1, 2). Sample SAS code is given below:

```
proc mixed data=use;
class year ;
model log30HzERGOU = year_numeric group1 group3 group4 year_times_group1 year_times_group3 year_times_group4 /solution;
random intercept /subject=person_id;
estimate "A effect" year_times_group1 0.5 year_times_group3 0.5 year_times_group4 -0.5  ;
estimate "E effect" year_times_group1 -0.5 year_times_group3 0.5 year_times_group4 0.5 ;
estimate "interaction" year_times_group1 1 year_times_group3 -1 year_times_group4 1 ;
```

The covariance matrix between study years was inspected and had smoothly decreasing values moving away from the diagonal with range 0.97-0.90, and therefore a random intercept model was used, rather than a repeated measures model with a structured covariance matrix.

For power calculations for longitudinal progression rates within gene subgroups, power was estimated using the sample size formula: $Z_{1-\beta} = \sqrt{\frac{N*\delta^2}{2*\sigma^2}} - Z_{1-\frac{\alpha}{2}}$, where β is the power corresponding to the z-score of a normal distribution, N is the total sample size, δ is the effect size for the yearly rates of change, σ^2 is the variance of the estimated slope which equals the sum of between-subject variability

and within-subject variability, α is the significance level which is calculated as 5%. The 2 in the denominator reflects the two comparison groups (with and without treatment), for similarly sized groups.

Rates of change are presented in loge units/year of remaining function. This rate represents the speed of the exponential decay. To convert to percent per year, there is an approximation (with <10% error for values less than -0.18 loge units) that, for example, -0.10 loge units/year = -10%/year. The accurate conversion formula is PercentPerYear = 100*(exp(LogsPerYear-1) . For example, using this formula, -0.18 loge units per year equals -16.5% per year of remaining function.

Longitudinal analyses in R

Analyses were performed in version 4.1.3 using the Lmer package. Sample R code is below. “Year” was the study year. “person_id” was the subject id. “Trt” was defined as a factor representing each of the four treatment groups; the reference group was A-/E- and the last three coefficients in the contrast coefficient vector below represent the interaction with year of the A+/E- , A+/E+, and A-/E+ treatment groups, respectively.

```
model = lmer(log30HzERGOU ~ trt*year + (1|person_id), dataset)
tA = glht(model, linfct = matrix(c(0, 0, 0, 0, 0, 0.5, 0.5, -0.5), 1))
tE = glht(model, linfct = matrix(c(0, 0, 0, 0, 0, -0.5, 0.5, 0.5), 1))
tI = glht(model, linfct = matrix(c(0, 0, 0, 0, 0, 1, -1, 1), 1))
summary(tA)
summary(tE)
summary(tI)
```

Gene-specific models were also created in R using a similar model. In the SAS analysis above and in the original study, the year variable had been rounded to the nearest integer. In the R lmer package, the rounding is not required. Rounded or unrounded year data gave nearly identical results (though small effects can cause crossing of the significance threshold for the borderline p-values, such as the *EYS* group - see Results). For the model using propensity score matching (Table 2), matching was implemented using the MatchIt package/function in R. For the outlier detection of baseline implicit time described in the text, boxplot.stats()$out was used to detect outliers.

Homogeneity testing

For homogeneity testing between the three clinical trial data sources, we assessed for homogeneity of the three data sets using the following linear regression model: Ln(ERG amplitude) = b_0 + b_1*treatment group + b_2*year + b_3*source + b_4*baseline implicit time + b_5*treatment group*year + b_6*source*year + b_7* baseline implicit time *year, where the b coefficients are fit by the regression model, and “source” is the variable representing one of three clinical trial data sources. The beta described in the text to determine homogeneity is b_6.

Supplementary Table 2

Gene	percent per year, this study	percent per year, prior studies	Reference
EYS	-16.0 ± 2.9		
PRPF31	-9.6 ± 2.2	-9.2	(3)
RHO	-6.8 ± 1.5	-8.7	(4)
RPGR[*]	-9.0 ± 1.8	-7.1	(5)
USH2A	-15.0 ± 1.5	-13.2	(6)

Supplementary Table 2. Progression rate of the 30 Hz cone flicker ERG amplitude of the largest gene groups, in percent per year of remaining function, ± 95% confidence interval of the mean. [*]The *RPGR* cohort was restricted to an RP phenotype in this cohort, but not in the prior study.

References for Supplement:

1. *Berson EL, Rosner B, Sandberg MA, Hayes KC, Nicholson BW, Weigel-DiFranco C, et al. A randomized trial of vitamin A and vitamin E supplementation for retinitis pigmentosa. Arch Ophthalmol. 1993;111(6):761-72.*
2. *Stampfer MJ, Buring JE, Willett W, Rosner B, Eberlein K, Hennekens CH. The 2 x 2 factorial design: its application to a randomized trial of aspirin and carotene in U.S. physicians. Stat Med. 1985;4(2):111-6.*
3. *Hafler BP, Comander J, Weigel DiFranco C, Place EM, Pierce EA. Course of Ocular Function in PRPF31 Retinitis Pigmentosa. Semin Ophthalmol. 2016;31(1-2):49-52.*
4. *Berson EL, Rosner B, Weigel-DiFranco C, Dryja TP, Sandberg MA. Disease progression in patients with dominant retinitis pigmentosa and rhodopsin mutations. Invest Ophthalmol Vis Sci. 2002;43(9):3027-36.*
5. *Sandberg MA, Rosner B, Weigel-DiFranco C, Dryja TP, Berson EL. Disease course of patients with X-linked retinitis pigmentosa due to RPGR gene mutations. Invest Ophthalmol Vis Sci. 2007;48(3):1298-304.*
6. *Sandberg MA, Rosner B, Weigel-DiFranco C, McGee TL, Dryja TP, Berson EL. Disease course in patients with autosomal recessive retinitis pigmentosa due to the USH2A gene. Invest Ophthalmol Vis Sci. 2008;49(12):5532-9.*

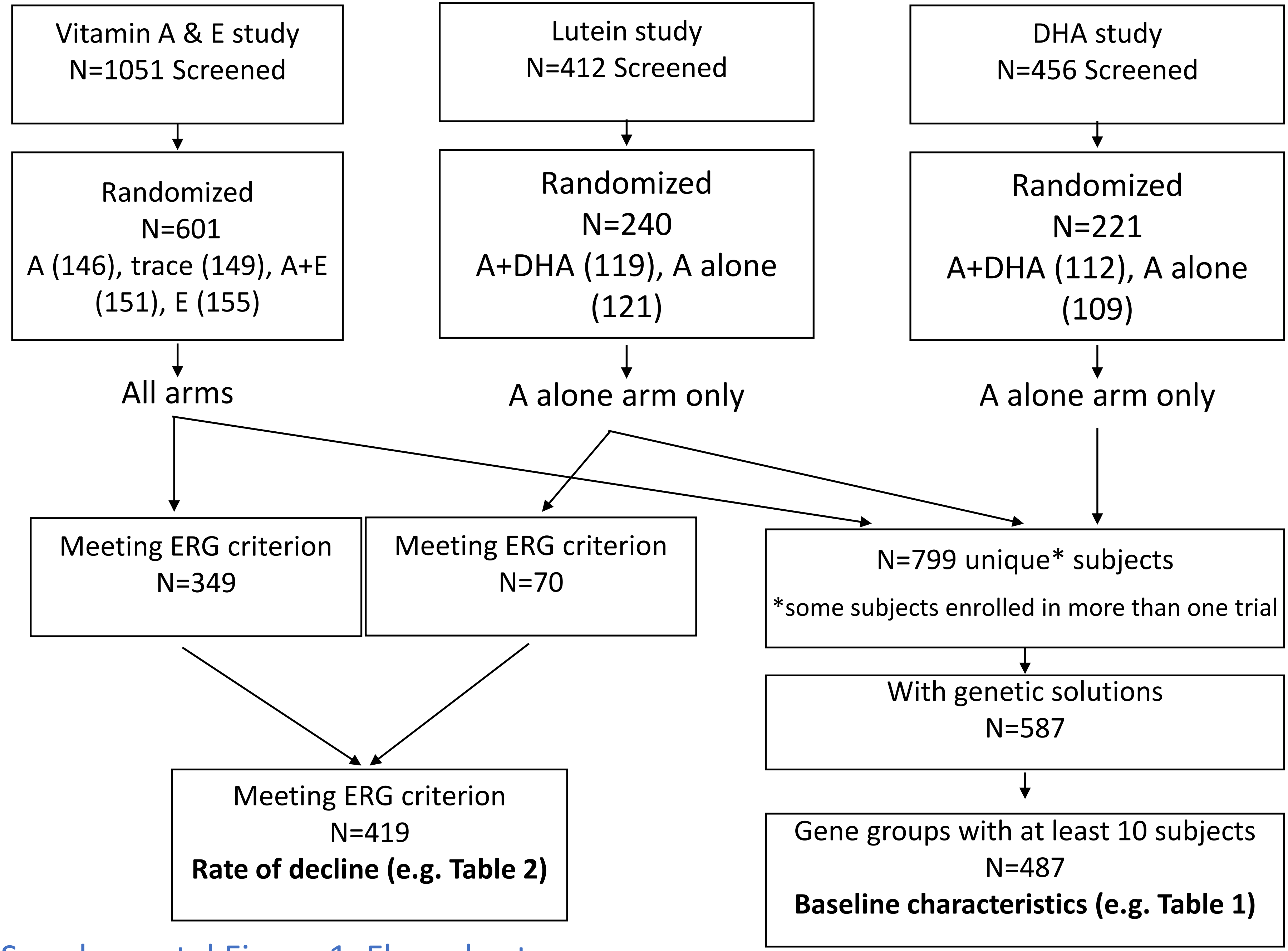

Supplemental Figure 1. Flow chart
